# Bedside assessment of regional cerebral perfusion using near-infrared spectroscopy and indocyanine green in patients with atherosclerotic occlusive disease

**DOI:** 10.1038/s41598-018-19668-5

**Published:** 2018-01-19

**Authors:** Hiroshi Saito, Tatsuya Ishikawa, Jun Tanabe, Shinya Kobayashi, Junta Moroi

**Affiliations:** 1 Department of Surgical Neurology, Research Institute for Brain and Blood Vessels-AKITA, Akita, Japan; 2 Department of Neurosurgery, Kameda medical center, Chiba, Japan

## Abstract

This pilot study aimed to investigate the utility of near-infrared spectroscopy/indocyanine green (NIRS/ICG) for examining patients with occlusive cerebrovascular disease. Twenty-nine patients with chronic-stage atherosclerotic occlusive cerebrovascular disease were included. The patients were monitored using NIRS at the bedside. Using ICG time-intensity curves, the affected-to-unaffected side ratios were calculated for several parameters, including the maximum ICG concentration (ΔICGmax), time to peak (TTP), rise time (RT), and blood flow index (BFI = ΔICGmax/RT), and were compared to the affected-to-unaffected side ratios of the regional cerebral blood flow (rCBF) and regional oxygen extraction fraction (rOEF) obtained using positron emission tomography with ^15^O-labeled gas. The BFI ratio showed the best correlation with the rCBF ratio among these parameters (r = 0.618; P = 0.0004), and the RT ratio showed the best correlation with the rOEF ratio (r = 0.593; P = 0.0007). The patients were further divided into reduced rCBF or elevated rOEF groups, and the analysis revealed significant related differences. The present results advance the measurement of ICG kinetics using NIRS as a useful tool for the detection of severely impaired perfusion with reduced rCBF or elevated rOEF. This method may be applicable as a monitoring tool for patients with acute ischemic stroke.

## Introduction

During acute cerebral stroke, the dynamics of cerebral circulation change continuously, either naturally or intentionally upon therapeutic interventions. Local cerebral circulation in patients with stroke has been evaluated using positron emission tomography (PET), single photon emission computed tomography (SPECT), CT perfusion imaging, and, recently, magnetic resonance (MR) perfusion imaging. However, it is impossible to perform these methods repeatedly in a timely manner during the acute phase of stroke. Moreover, the patients require transportation to each laboratory to undergo such examinations. Therefore, it is desirable to establish a convenient bedside examination method that has an acceptable time requirement, and is capable of detecting changes in local cerebral circulation dynamics in the acute phase of stroke in a clinical setting.

The evaluation of cerebral circulation with near-infrared spectroscopy (NIRS) and intravenous administration of indocyanine green (ICG) as an intravascular tracer (NIRS/ICG) was first reported by Kuebler *et al*.^1^. They reported that the blood flow index (BFI) significantly correlated with the regional cerebral blood flow (rCBF). Later, parameters such as the time to peak (TTP), rise time (RT), and mean transit time (MTT) calculated from the ICG time concentration curve have been advocated to evaluate local cerebral circulation^2–7^. However, the clinical significance and utility of such parameters have not been well understood.

Currently the NIRS/ICG approach is considered a useful tool for evaluating the dynamics of cerebral circulation in patients in the acute stage of stroke in a clinical setting. However, it has not been clear whether cerebral circulation can be appropriately evaluated with NIRS/ICG. In the present preliminary study, we examined cerebral circulation and metabolism in patients with cerebral ischemia in the chronic stage both by ^15^O-labeled gas PET and NIRS/ICG. We defined several parameters such as ΔICGmax (the maximum ICG concentration), TTP (the time between 0 and 100% of the maximum ICG concentration), RT (the time between 10 and 90% of the maximum ICG concentration), and BFI (=ΔICGmax/RT) and normalized the values from the affected side to unaffected side. We specifically analyzed how these parameters obtained using NIRS/ICG are correlated with the measured values obtained using PET, which has been considered the gold standard in the evaluation of cerebral circulation and metabolism.

## Results

### Can NIRS/ICG safely be performed at the bedside?

In all the thirty-one patients who underwent a bedside NIRS/ICG examination, no side effects appeared to be associated with the examination. We excluded two patients from the study, one who had experienced extensive cerebral infarction, and another for whom the ICG time-concentration curve could not be generated.

### Do PET parameters have any correlation with NIRS/ICG measures?

**Figure 1:**
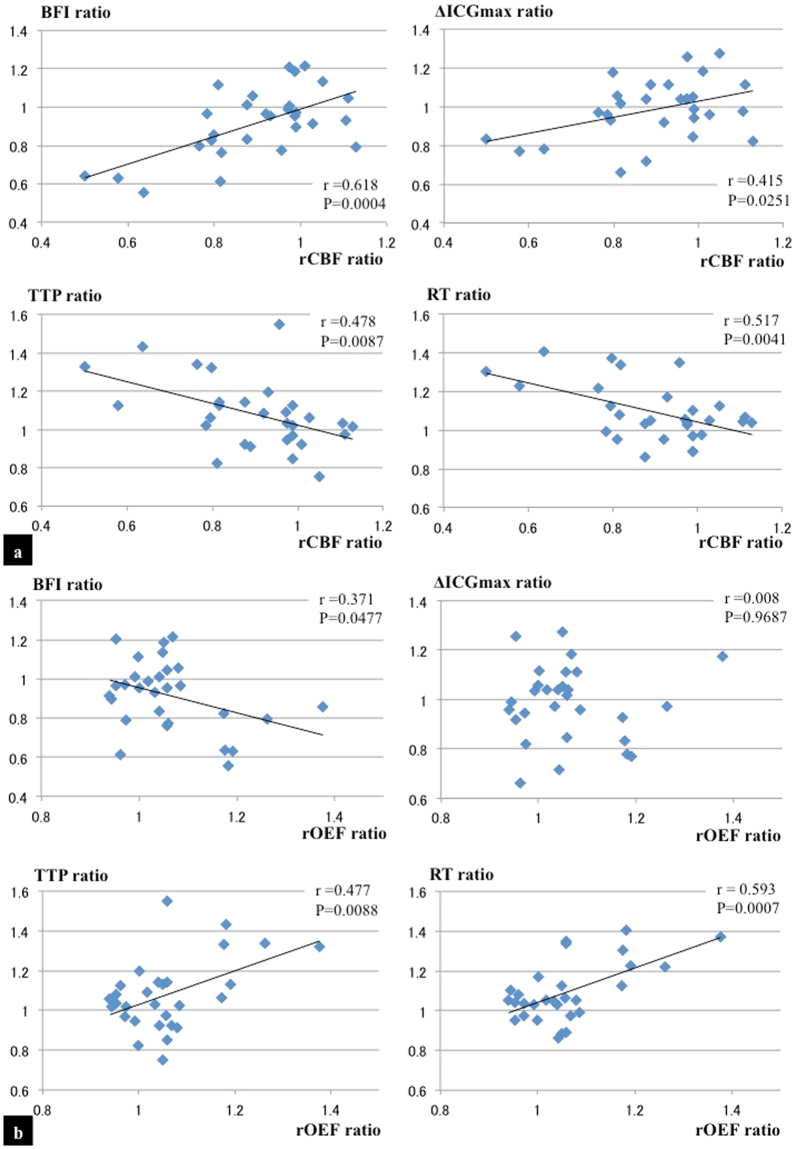
Correlations between the rCBF ratio and several NIRS/ICG parameters (BFI ratio, ΔICGmax ratio, TTP ratio, and RT ratio) are shown (**a**). Correlations between the rOEF ratio and NIRS/ICG parameters are also demonstrated (**b**). Among the parameters of the NIRS/ICG, the BFI ratio shows the best correlation with the rCBF ratio (|r| = 0.618, P = 0.0004), and the RT ratio shows the best correlation with the rOEF ratio (|r| = 0.593, P = 0.0007).

Twenty-nine patients were included in the study. The patient profiles are shown in Table 1. One patient had bilateral stenosis in the internal carotid artery (ICA) and the symptomatic side was considered the affected side. There were significant differences between the control group and the patients with stroke group in the male:female ratio and ratio of having diabetes mellitus. Figure 1 depicts the relationship between the NIRS/ICG parameters (ΔICGmax, TTP, RT, and BFI ratio) and the PET parameters (rCBF and regional oxygen extraction fraction [rOEF] ratios). Among the NIRS/ICG parameters, the BFI ratio showed the best correlation with the rCBF ratio (r = 0.618, P = 0.0004; Fig. 1a), while the RT ratio showed the best correlation with the rOEF ratio (r = 0.593, P = 0.0007; Fig. 1b).

**Table 1:** Characteristics of the study participants.

	Control (N = 12)	Patients with stroke (N = 29)
Age (years)	62.3 ± 11.6	63.7 ± 9.2
Sex, male	3 (25%)	24 (83%)*
Side (Left)	Not available	17 (59%)
Location of occlusion or severe stenosis		
ICA M1	Not available	20 (69%)
	Not available	9 (31%)
Symptom	Not available	21 (72%)
Smoking	2 (17%)	9 (31%)
Hypertension	7 (58%)	22 (76%)
Diabetes mellitus	1 (17%)	13 (45%)*
Atrial fibrillation	0 (0%)	2 (7%)

As a control for the NIRS/ICG measures, preoperative and asymptomatic patients who were scheduled to undergo craniotomy for their unruptured cerebral aneurysms were selected. These patients had neither a history of cerebral strokes nor stenosis of more than 50% on the preoperative MR angiography.

Values are given as an average ± standard deviation or as number (%). *Significantly different by Fisher’s exact test (P < 0.05). One patient had bilateral stenosis in the ICA.

ICA, the internal carotid artery; M1, horizontal portion of the middle cerebral artery.

**Figure 2:**
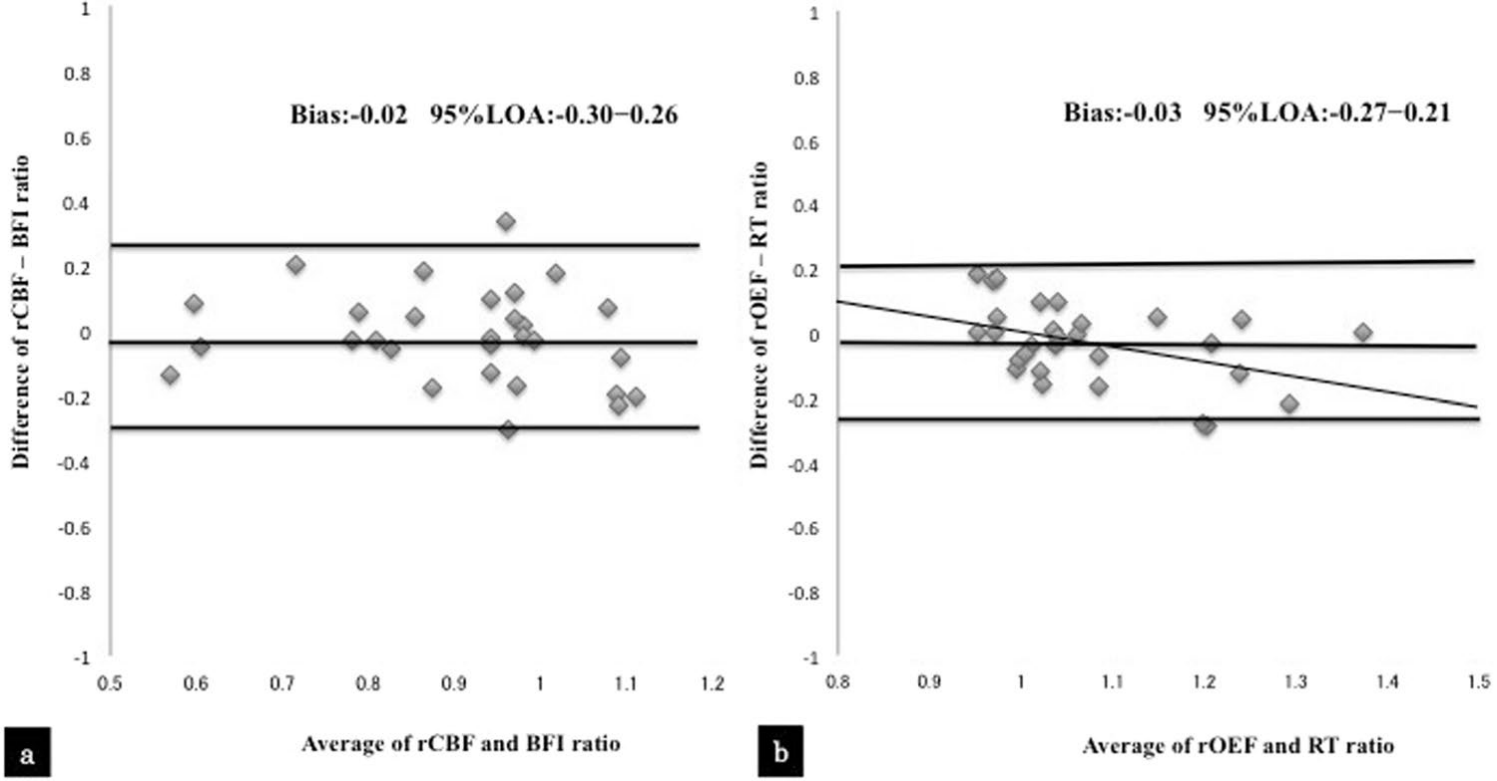
Brand-Altman analysis of the difference between the rCBF and BFI ratios *versus* the average of the rCBF and BFI ratios (**a**). Brand-Altman analysis of the difference between the rOEF and RT ratios *versus* the average of the rOEF and RT ratios (**b**).

Figure 2 shows a Bland-Altman plot for the two methods. As shown in Fig. 2a, there was no fixed bias or proportional bias in the difference between the rCBF and BFI ratios *versus* the average of the rCBF and BFI ratios. The mean bias was −0.02, and the 95% limits of agreement were −0.30 to 0.26. Figure 2b shows that there was no fixed bias in the difference between the rOEF and RT ratios *versus* the average of the rOEF and RT ratios. The mean bias was −0.03, and the 95% limits of agreement were −0.27 to 0.21. As shown in the regression line of the difference *versus* average of the two methods, a proportional bias did exist.

### Do NIRS/ICG measures detect impaired cerebral blood flow and oxygen metabolism?

In the gas PET study, seven patients were assigned to the reduced rCBF group, and six were included in the elevated rOEF group. The two groups had six patients in common. We compared the NIRS/ICG parameters among the normal rCBF, reduced rCBF, and control groups (only in the NIRS/ICG protocol). There was no significant difference in the ΔICGmax ratio among the groups (control group, 1.08 ± 0.18; normal rCBF group, 1.01 ± 0.15; reduced rCBF group, 0.92 ± 0.14; P = 0.1109). The BFI ratio showed a significant difference between the normal rCBF group (0.97 ± 0.15) and the reduced rCBF group (0.75 ± 0.15; P = 0.0213), but not between the control group (1.09 ± 0.21) and the normal rCBF group (P = 0.1455; Table 2).

**Table 2:** rCBF and measured values of parameters by NIRS/ICG.

Ratio	Control (a) (N = 12)	Normal rCBF (b) (N = 22)	Reduced rCBF (c) (N = 7)	P value	
				a *versus* b	b *versus* c
ΔICG max	1.08 ± 0.18	1.01 ± 0.15	0.92 ± 0.14	0.1109	
TTP	0.97 ± 0.08	1.03 ± 0.16	1.23 ± 0.16	0.5205	0.0093*
RT	1.00 ± 0.09	1.05 ± 0.12	1.24 ± 0.14	0.4826	0.0034*
BFI	1.09 ± 0.21	0.97 ± 0.15	0.75 ± 0.15	0.1455	0.0213*

Ratio is given as affected side to non-affected side ratio.

Values are given as mean ± standard deviation. *A P value < 0.05 was defined as statistically significant.

BFI, blood flow index; ICG, indocyanine green; NIRS, near-infrared spectroscopy; rCBF, regional cerebral blood flow; RT, rise time; TTP, time to peak.

When we categorized the patients based on the rOEF values and analyzed the data among the groups, the RT ratios showed a significant difference between the elevated rOEF group (1.28 ± 0.11) and the normal rOEF group (1.05 ± 0.12; P = 0.0003), but not between the control group (1.00 ± 0.09) and normal rOEF group (P = 0.4646). Similarly, there was no significant difference between the control group (0.97 ± 0.08) and the normal rOEF group (1.03 ± 0.16) in the TTP ratio (P = 0.4965), but a significant difference in the discrimination level was observed between the elevated rOEF group and the other groups (1.27 ± 0.14; P = 0.0025; Table 3).

**Table 3:** rOEF and measured values of parameters by NIRS/ICG.

Ratio	Control (a) (N = 12)	Normal rOEF (b) (N = 23)	Elevated rOEF (c) (N = 6)	P value	
				a *versus* b	b *versus* c
ΔICG max	1.08 ± 0.18	1.01 ± 0.15	0.91 ± 0.15	0.1122	
TTP	0.97 ± 0.08	1.03 ± 0.16	1.27 ± 0.14	0.4965	0.0025*
RT	1.00 ± 0.09	1.05 ± 0.12	1.28 ± 0.11	0.4646	0.0003*
BFI	1.09 ± 0.21	0.97 ± 0.15	0.72 ± 0.12	0.1282	0.0086*

Ratio is given as affected side to non-affected side ratio.

Values are given as mean ± standard deviation. *A P value < 0.05 was defined as statistically significant.

BFI, blood flow index; ICG, indocyanine green; NIRS, near-infrared spectroscopy; rOEF, regional oxygen extraction fraction; RT, rise time; TTP, time to peak.

**Figure 3:**
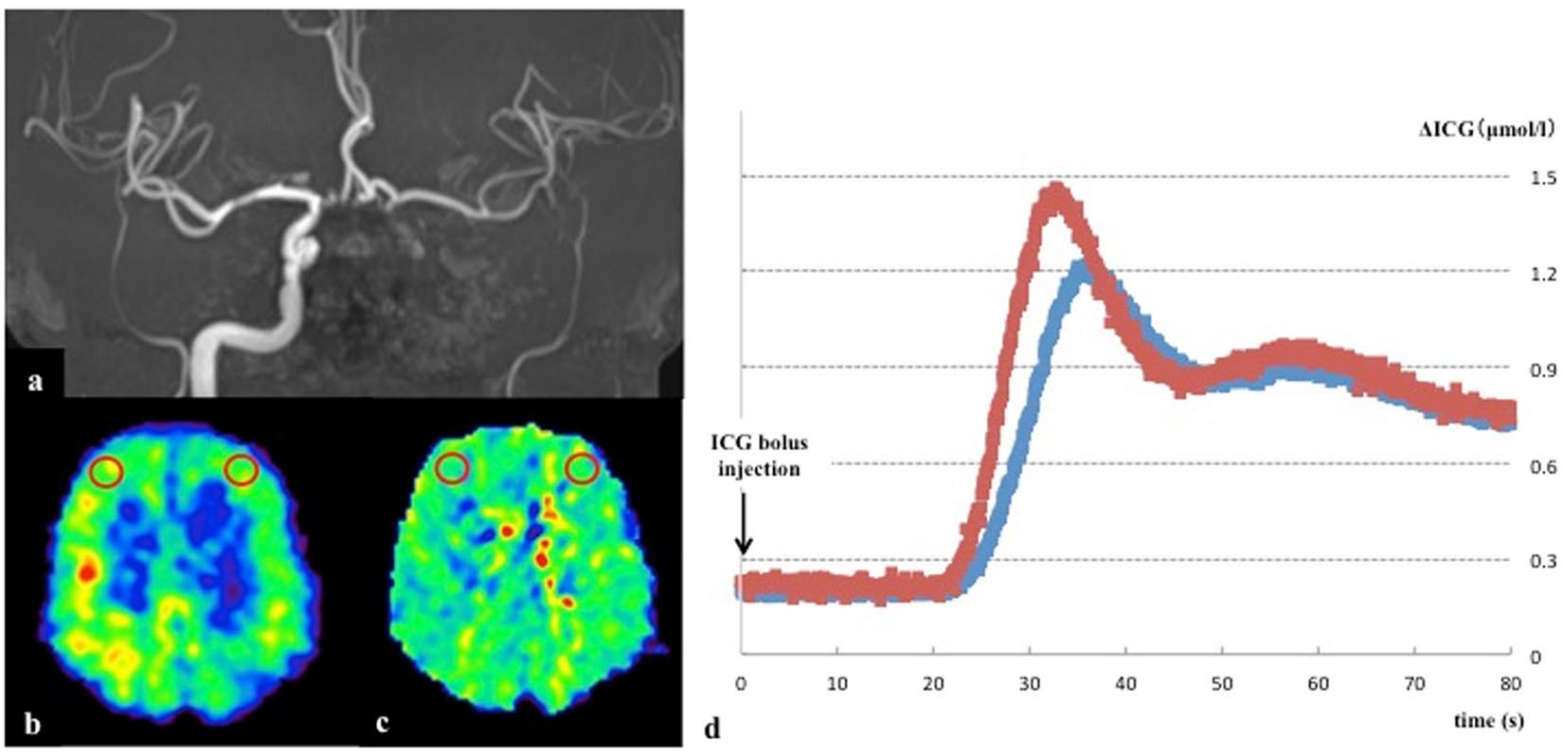
A representative case. A 66-year-old male patient who experienced a small cerebral infarction in the left frontal lobe and was found to have an occlusion of the left internal carotid artery by MRA (**a**). The ^15^O-labelled gas PET study revealed a reduced rCBF (rCBF ratio was 0.58) (**b**) and an elevated rOEF (rOEF ratio was 1.18) (**c**) in the target regions of interest (red circles). (**d**) The NIRS/ICG measurement provided an ICG time-intensity curve from the left site (blue line) as well as the right site (red line). the calculated ΔICGmax ratio was 0.83, TTP ratio was 1.10, the RT ratio was 1.30, and the BFI ratio was 0.64.

As an illustrative case, we present a 66-year-old male patient who was admitted to our hospital with a small cerebral infarction on the left frontal lobe. MR angiography (MRA) demonstrated occlusion of the left internal carotid artery (Fig. 3a). NIRS/ICG and PET examinations were performed approximately 3 weeks after the onset of the condition. The PET examination demonstrated a severely decreased CBF (rCBF ratio 0.58) and elevated OEF (rOEF ratio 1.18) of the left hemisphere in target ROIs (Fig. 3b,c); thus, the patient was included in the reduced rCBF group and elevated rOEF group. The NIRS/ICG measurement showed a time-intensity curve from the left site (blue line) and the right site (red line) of the NIRS optodes (Fig. 3d). In particular, the RT ratio (1.30) was markedly increased and the BFI ratio (0.64) was decreased.

## Discussion

The present study is the first report in which measures of PET are compared to parameters evaluated by NIRS/ICG in patients with cerebral ischemia. The ICG has absorption characteristics in the near infrared region with a peak at 805 Hz, and hemoglobin also has light absorption characteristics in this wavelength region. Therefore, after intravenous injection of ICG, a time concentration curve reflecting an increase in the ICG concentration in brain tissue via NIRS could be obtained. Several parameters can be calculated from such a time concentration curve. In a previous study, the BFI was reported to be significantly correlated with the blood flow measured by laser Doppler in a piglet model^1^. Similarly, in the present study, BFI was the parameter that showed the best correlation with rCBF. Its usefulness has been reported in patients with acute ischemic stroke and congenital heart disease, as well as in cerebral blood flow monitoring during carotid endarterectomy^2,5–8^. It has been previously reported that TTP, RT, and BFI significantly change in patients with cerebral infarction, and that the TTP measures obtained from NIRS/ICG test strongly correlated with those obtained by MR perfusion imaging^6,7^. Oldag *et al*.^5^ have reported that TTP measured by NIRS/ICG in patients with stenosis or occlusion of the MCA was prolonged and showed a good correlation with TTP measured by MR perfusion imaging. Based on these results, ICG is thought to exhibit hemodynamics similar to those of magnetic resonance imaging (MRI) and CT in terms of intravascular tracer, and may represent a useful method for evaluating the local cerebral blood flow dynamics.

In our patients, parameters such as BFI, RT, and TTP ratios showed significant differences between the affected and unaffected hemispheres, whereas the ΔICGmax ratio related to intracranial ICG concentrations failed to demonstrate a significant difference. In the report by Terberg *et al*.^7^, ΔICGmax showed a tendency to decrease in the side of the middle cerebral artery infarction as compared to the healthy side, although not significantly. When ICG is administered intravenously, it becomes diluted in the systemic circulation and reaches the target area at low concentrations. Therefore, semi-quantitative evaluations using the left-right ratio are not sensitive enough to evaluate the difference in the concentration gradient. Measurements related to ICG concentration are also affected by several other factors, such as the light volume in the room where the NIRS/ICG is undertaken. Similar effects can occur during the BFI measurement; however, its calculation includes RT as the time parameter, and the influence is thus conceivably smaller than that of the ΔICGmax value.

Our study participants were patients who had occlusion or severe stenosis of the internal carotid artery (ICA) or the middle cerebral artery (M1) and were diagnosed with possible hemodynamic ischemia. Patients whose brain tissue is exposed to severely disturbed cerebral perfusion with reduced rCBF, but who survive the injury without substantially developing cerebral infarction as measured by elevated rOEF, in other words Powers’ stage II^9^, would definitely benefit from vasoreconstructive interventions in the acute stage of cerebral ischemia. Although it is still unclear whether an NIRS/ICG examination can be applied for cerebral circulation monitoring in acute stroke patients, our results validate that a hemodynamically compromised condition where the oxygen carriage reserve is mobilized (i.e., misery perfusion^10^) can be detected by NIRS/ICG examination by detecting decreased BFI or increased RT ratios. However, it may be difficult to capture the stage of mild cerebral ischemia where only the vascular reserve is mobilized and not accompanied by delayed circulation. Furthermore, the Brand-Altman analysis showed a wide limit of agreement between the BFI and rCBF ratios (−0.30 to 0.26), and between the RT and OEF ratios (−0.27 to 0.21). Additionally, there was a proportional bias between the RT and OEF ratios. These findings suggest that the RT ratio may overestimate the degree of poor cerebral perfusion. On the other hand, OEF measured by PET has previously been reported to correlate with MTT/CT perfusion as well as MTT/MR perfusion when using other kinds of intravascular tracer^11,12^. The present study suggested that parameters measured by NIRS/ICG test could show the characters of misery perfusion (both decreased rCBF and increased rOEF) indicated by ^15^O-labeled gas PET parameters in patients with ipsilateral hemodynamic impairment. In acute atherothrombotic stroke, cerebral ischemia is mainly characterized by a marked increase in OEF rather than mild increase of cerebral blood volume (CBV)^13^. Thus, acute misery perfusion could easily occur in acute atherothrombotic stroke. Further, the reduction of rCBF could strongly correlate with the prolonged transit time in acute stroke. Therefore, our findings suggest that the BFI and RT ratios obtained using NIRS/ICG may be valuable bed-side monitoring tools for patients with acute ischemic stroke. Indeed, several small studies have reported the usefulness of NIRS/ICG test in acute stroke^6,7,14^. However, to validate its usefulness, it will be necessary to perform large prospective studies in the future.

Additionally, we did not recognize any side effects associated with the examination in this series of patients, in contrast to previous evidence of a minimal risk of neurotoxicity in two previous studies^15,16^. Nevertheless, we suggest that the NIRS/ICG test is a very safe test that can easily be performed even on the bedside, and that the dynamics of ICG measured via NIRS can be taken to reflect the local cerebral circulation.

One major methodological problem of this approach is the limited spatial resolution of NIRS. The measures represent only the values in the area beneath the optodes. Multi-channel NIRS measurements expand the measurable area^5^, but cannot measure cerebral tissue that is deeper than 3 cm below the optodes. However, if the occlusive cerebrovascular disease also involves a major trunk artery, such as the ICA or M1, then the measures from the forehead optodes may adequately represent the hemispheric cerebral circulation in clinical settings.

There are several additional methodological limitations in the present findings. First, the size of the study population was too small to draw strong conclusions. In particular the number of patients within the elevated rOEF group and reduced rCBF group was relatively small. Second, the rCBF has been shown to be influenced by the PaCO_2_ and hemoglobin concentrations^17–20^; however, we did not examine any of these parameters when we measured the ICG time-intensity curve. Additionally, the skin blood flow has been found to contaminate cerebral NIRS measurements^21^. However, its influence on the ICG kinetics seems to be limited: in a piglet model, no correlation was found between the blood flow index and skin blood flow^1,22^. We have attempted to exclude these factors by comparing them using the affected-to-unaffected side ratio, but this approach will not work when evaluating patients with bilateral stenosis or patients with mild cerebral ischemia.

## Conclusions

The present study showed that semi-quantitative ICG assessment by means of NIRS at the bedside (in particular, the RT and BFI ratios) is a useful tool for detection of hemodynamically compromised conditions where the oxygen carriage reserve is mobilized, i.e., misery perfusion, by stenosis and occlusion of the ICA or M1. Furthermore, this technique is easy to apply at the bedside, and can be repeated without serious side effects.

## Methods

### Patient selection

We examined thirty-one patients with atherosclerotic occlusive cerebrovascular disease, admitted to our hospital from April 2013 to March 2016, regarding their cerebral perfusion and metabolism, both by gas PET and NIRS/ICG. We assessed twenty-nine patients (average age 63.7 years, twenty-four male patients) excluded two patients (one patient had experienced extensive cerebral infarction, and another patient for whom the ICG time-concentration curve could not be generated). The patients had an occlusion or severe stenosis (more than 70%) of the ICA or the horizontal portion of M1 as evidenced by MRA. Many were symptomatic patients who had developed cerebral infarction or transient ischemic attacks, but the rest were asymptomatic, being incidentally diagnosed with vascular lesions. In the symptomatic patients, both PET and NIRS/ICG examinations were performed no earlier than 3 weeks after stroke onset. The NIRS/ICG was performed on the same day or within several days of the gas PET. When the hemispheric side of the cerebral ischemia and vascular lesion were consistent, we identified that as the affected side. When the patients had bilateral vascular lesions, the side where the symptomatic cerebral ischemia occurred was regarded as the affected side. In the asymptomatic patients, we identified the affected side as the side where the vascular lesions were located.

All patients signed informed consent forms approved by the institutional review boards. Ethical approval was provided by the Institutional Review Board for Research Institute for Brain and Blood Vessels-AKITA (Research Number 12-13). All methods were performed in accordance with approved guidelines and regulations.

### Gas PET examination (Gas PET protocol)

The ^15^O-labeled Gas PET study was performed with a three-dimensional (3D) PET scanner (SET-3000GCT/M; Shimadzu Corp, Kyoto, Japan). In our institution, three static emission scans with inhalation of C ^15^O, inhalation of ^15^O_2_, and injection of H_2_^15^O were performed to examine the rCBF, rCBV, rOEF and regional cerebral metabolic rate of oxygen, respectively, as described previously^23,24^. The interval between scans was approximately 15 min. Continuous arterial blood sampling was conducted throughout the PET scanning by using a catheter implanted in the radial artery to measure the arterial isotope activities. Blood pressure was recorded for each PET measurement and used to assess changes in mean blood pressure. PaCO_2_, PaO_2_, and blood pH were also measured in the same samples.

**Figure 4:**
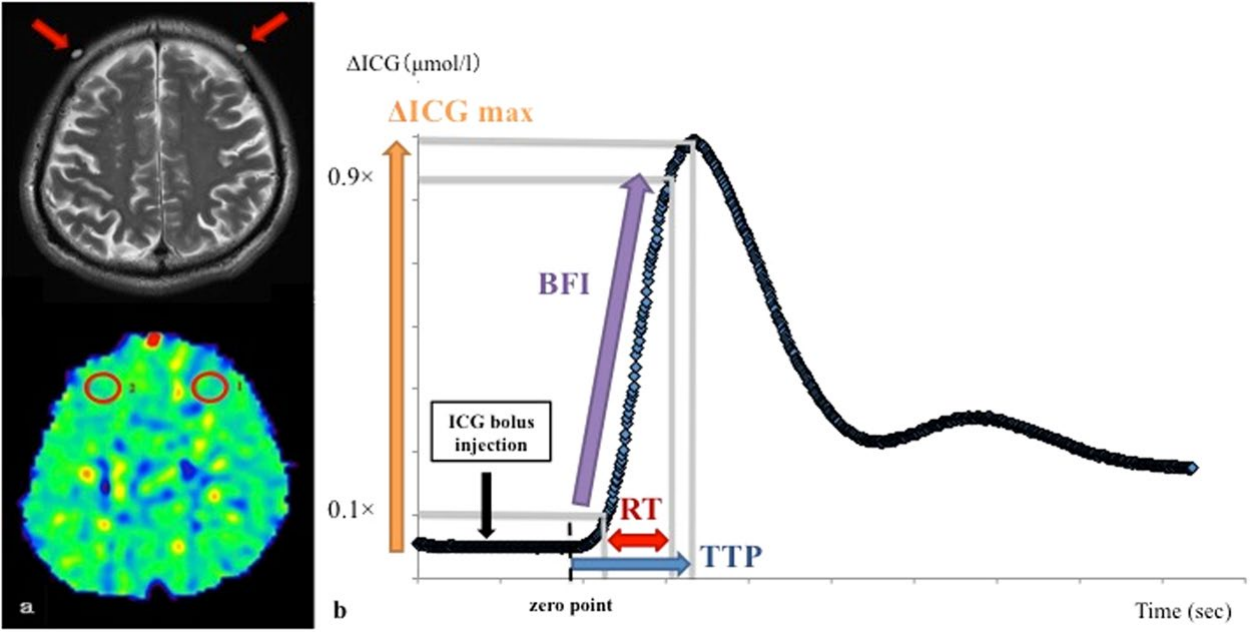
The arrow indicates the site of the optodes for the NIRS procedure on an MRI T2 weighted image (**a**, top). The circular regions of interest were placed manually at the site presumed to be measured by NIRS, and rCBF and rOEF were measured on the result of a ^15^O-labeled gas PET study (**a**, bottom). A representative ICG time-intensity curve showing the parameters used to estimate the regional cerebral perfusion. We set the zero point as the time to rise to +4 standard deviation of the average ICG concentration in the period 0–10 seconds after intravenous injection, and calculated ΔICGmax (the maximum ICG concentration), TTP (time to peak: the time between 0 and 100% of the maximum ICG concentration), RT (rise time: the time between 10 and 90% of the maximum ICG concentration), and BFI(blood flow index: ΔICGmax / RT). Each measured parameter was normalized using the affected-to-unaffected side ratio (**b**).

According to a previous report, NIRS is believed to reflect the hemodynamics of the cerebral tissue at 3 cm below the optodes or at 0.9 cm below the brain surface^25^. Therefore, for the PET examination a circular region of interest of 16 mm diameter was placed to cover the area presumed to be measured by NIRS, and the rCBF and rOEF were measured (Fig. 4a).

Based on the result of the gas PET study, the patients were divided into two groups: a reduced rCBF group (n = 7), which included the patients with an rCBF affected-to-unaffected side ratio of less than 0.8, and a normal rCBF group (n = 22), which included the remaining patients. They were additionally grouped into a normal rOEF group (n = 23) and an elevated rOEF group (n = 6). The latter included the patients with affected-to-unaffected ratio of rOEF of 1.13 or more, while the remaining patients were included in the normal rOEF group. We opted this value of rOEF, 1.13, on the basis of the COSS study^26^.

### Measurement of ICG concentration by NIRS (NIRS/ICG protocol)

ICG time-intensity curves were measured using NIRS (NIRO-200 NX, Hamamatsu Photonics, Japan) with intravenous administration of ICG at 0.1 mg/kg. The NIRS provides light in the infrared region at three distinct wavelengths (735, 810, and 850 nm) and the receiving probe collected the scattered light with a sampling rate of 20 Hz. The absolute changes in ICG concentration were calculated based on the near infrared light attenuation according to the modified Beer-Lambert law using a specific software package (Hamamatsu Photonics). The NIRS optodes were placed bilaterally on the forehead with the center of the optodes on the scalp approximately 3 cm above the eyebrows, and a lateral interoptode distance of 4 cm to cover the bilateral frontal watershed area between the anterior and middle cerebral arteries. The optodes were fixed by an elastic band and covered with light-shielding cloth. These measurements were performed at the bedside in the ward.

We set the zero point as the time to rise to +4 standard deviations (SD) of the average ICG concentration in the period 0–10 s after the intravenous injection, and calculated the ΔICGmax (the maximum ICG concentration), the TTP (defined as the time between 0 and 100% of the maximum ICG concentration), the RT (defined as the time between 10 and 90% of the maximum ICG concentration), and the BFI (blood flow index = ΔICGmax/RT) from the ICG time-density curve (Fig. 4b). Each measured parameter was normalized using the affected-to-unaffected side ratio.

For the normal control NIRS/ICG measures, we conducted NIRS/ICG tests on 12 patients (average age 62.3 years, three male patients) who did not have a history of any cerebral stroke and were in a preoperative state of craniotomy for unruptured cerebral aneurysms (Table 1). These control patients were confirmed not to have a stenosis of more than 50% in the cerebral artery by preoperative angiography (3D CT angiography and/or MRA). These measures were used as “control values” for each parameter in the NIRS/ICG anaylsis.

### Statistics

All values are given as means ± SD. The controls were compared with the study participants using Fisher’s exact test. The relations between the parameters of the NIRS/ICG method and those of the gas PET were evaluated with linear regression analysis. Correlations between the NIRS/ICG and the PET parameters were determined by Pearson’s correlation coefficient. One-way analysis of variance (ANOVA) was used for comparing the results among groups, with a Scheffe *post-hoc* test for multiple comparisons. A Bland-Altman plot^27^ was constructed to evaluate correlations between the measures derived from two methods. For all tests, a P-value < 0.05 indicated a statistically significant difference.

### Data availability

Data of the current study is available from the corresponding author on reasonable request.

## References

[1] Kuebler WM (1998). Noninvasive measurement of regional cerebral blood flow by near-infrared spectroscopy and indocyanine green. J. Cereb. Blood Flow Metab..

[2] Kato S, Yoshitani K, Ohnishi Y (2016). Cerebral blood flow measurement by near-infrared spectroscopy during carotid endarterectomy. J. Neurosurg. Anesthesiol..

[3] Keller E (2003). Noninvasive measurement of regional cerebral blood flow and regional cerebral blood volume by near-infrared spectroscopy and indocyanine green dye dilution. NeuroImage.

[4] Kobayashi S, Ishikawa T, Tanabe J, Moroi J, Suzuki A (2014). Quantitative cerebral perfusion assessment using microscope-integrated analysis of intraoperative indocyanine green fluorescence angiography versus positron emission tomography in superficial temporal artery to middle cerebral artery anastomosis. Surg. Neurol. Int..

[5] Oldag A (2012). Assessment of cortical hemodynamics by multichannel near-infrared spectroscopy in steno-occlusive disease of the middle cerebral artery. Stroke.

[6] Terborg C, Bramer S, Harscher S, Simon M, Witte OW (2004). Bedside assessment of cerebral perfusion reductions in patients with acute ischaemic stroke by near-infrared spectroscopy and indocyanine green. J. Neurol. Neurosurg. Psychiatry.

[7] Terborg C (2009). Noninvasive assessment of cerebral perfusion and oxygenation in acute ischemic stroke by near-infrared spectroscopy. Eur. Neurol..

[8] Wagner BP, Gertsch S, Ammann RA, Pfenninger J (2003). Reproducibility of the blood flow index as noninvasive, bedside estimation of cerebral blood flow. Intensive Care Med..

[9] Powers WJ (1991). Cerebral hemodynamics in ischemic cerebrovascular disease. Ann. Neurol..

[10] Baron JC (1981). Reversal of focal “misery-perfusion syndrome” by extra-intracranial arterial bypass in hemodynamic cerebral ischemia. A case study with ^15^O positron emission tomography. Stroke.

[11] Kajimoto K (2003). Cerebral hemodynamic evaluation using perfusion-weighted magnetic resonance imaging: comparison with positron emission tomography values in chronic occlusive carotid disease. Stroke.

[12] Kamath A (2008). Perfusion CT compared to H(2) (15)O/O (15)O PET in patients with chronic cervical carotid artery occlusion. Neuroradiology.

[13] Heiss WD (1992). Progressive derangement of periinfarct viable tissue in ischemic stroke. Journal of cerebral blood flow and metabolism. J. Cereb. Blood Flow Metab..

[14] Steinkellner O (2010). Optical bedside monitoring of cerebral perfusion: technological and methodological advances applied in a study on acute ischemic stroke. J. Biomed. Optics.

[15] Keller E (2002). Evaluation of brain toxicity following near infrared light exposure after indocyanine green dye injection. J. Neurosci. Methods.

[16] Toczylowska B (2014). Neurotoxic effects of indocyanine green -cerebellar granule cell culture viability study. Biomed. Opt. Express.

[17] Ibaraki M (2010). Interindividual variations of cerebral blood flow, oxygen delivery, and metabolism in relation to hemoglobin concentration measured by positron emission tomography in humans. J. Cereb. Blood Flow Metab..

[18] Reivich M (1964). Arterial Pco2 and cerebral hemodynamics. Am. J. Physiol..

[19] Scheinberg P (1951). Cerebral blood flow and metabolism in pernicious anemia. Blood.

[20] Thomas DJ (1977). Cerebral blood-flow in polycythaemia. Lancet.

[21] Smielewski P, Czosnyka M, Pickard JD, Kirkpatrick P (1997). Clinical evaluation of near-infrared spectroscopy for testing cerebrovascular reactivity in patients with carotid artery disease. Stroke.

[22] Harris DN, Cowans FM, Wertheim DA (1994). NIRS in the temporal region Strong influence of external carotid artery. Adv. Exp. Med. Biol..

[23] Hatazawa J (1995). Regional cerebral blood flow, blood volume, oxygen extraction fraction, and oxygen utilization rate in normal volunteers measured by the autoradiographic technique and the single breath inhalation method. Ann. Nucl. Med..

[24] Ibaraki M (2008). Quantification of cerebral blood flow and oxygen metabolism with 3-dimensional PET and 15O: validation by comparison with 2-dimensional PET. J. Nucl. Med..

[25] Hock C (1997). Decrease in parietal cerebral hemoglobin oxygenation during performance of a verbal fluency task in patients with Alzheimer’s disease monitored by means of near-infrared spectroscopy (NIRS) — correlation with simultaneous rCBF-PET measurements. Br. Res..

[26] Powers WJ (2011). Extracranial-intracranial bypass surgery for stroke prevention in hemodynamic cerebral ischemia: the Carotid Occlusion Surgery Study randomized trial. JAMA.

[27] Bland JM, Altman DG (1986). Statistical methods for assessing agreement between two methods of clinical measurement. Lancet.

